# Multiple eumenorrheic cycles are necessary to observe a significant increase in estrogen exposure and ovulation in exercising women with functional hypothalamic oligo/amenorrhea undergoing a nutritional intervention: Insights from the REFUEL study

**DOI:** 10.1002/pmrj.70024

**Published:** 2025-10-16

**Authors:** Rebecca J. Mallinson, Nancy I. Williams, Emily A. Ricker, Heather C. M. Allaway, Mary Jane De Souza

**Affiliations:** ^1^ Department of Kinesiology Penn State Harrisburg Middletown Pennsylvania USA; ^2^ Department of Kinesiology Pennsylvania State University University Park Pennsylvania USA; ^3^ Consortium for Health and Military Performance, Department of Military and Emergency Medicine, F. Edward Hébert School of Medicine Uniformed Services University Bethesda Maryland USA; ^4^ Henry M Jackson Foundation for the Advancement of Military Medicine, Inc.; ^5^ School of Kinesiology Louisiana State University Baton Rouge Louisiana USA

## Abstract

**Background:**

Due to consequences of energy‐related oligo‐/amenorrhea (Oligo/Amen) among exercising females, recovery of menses (ROM) is a priority. ROM is inconsistently defined and rarely reported with reproductive hormone (estrogen, progesterone) data, making it difficult to know when females achieve adequate recovery.

**Objective:**

The purpose of this secondary analysis of the REFUEL randomized controlled trial was to explore the ovarian hormone environment and quality of menstrual recovery among varying ROM definitions.

**Methods:**

ROM was assessed in exercising females with Oligo/Amen (*n* = 33) who participated in a 12‐month intervention of increased energy intake. Four ROM definitions (onset of menses, 1 menstrual cycle <36 days, 2 cycles <36 days, or 3 cycles <36 days) demonstrating advancing degrees of recovery were evaluated. Urinary metabolites of estrogen (estrone‐1‐glucuronide) and progesterone (pregnanediol glucuronide) were measured daily in a baseline and recovery menstrual cycle; the change in ovarian hormone exposure from baseline to recovery menstrual cycle was analyzed for each ROM definition. The proportion of ovulatory versus anovulatory recovery cycles and the proportion of exercising females who experienced a relapse of Oligo/Amen post recovery were calculated.

**Results:**

During the intervention, 58% (19/33) of females satisfied at least one ROM definition. There was no change in average ovarian hormone exposure from baseline to the recovery cycle until females experienced three consecutive cycles <36 days, when estrogen exposure significantly increased (+154.7 ng/mL*day, +32.5%, *p* < .04). As females achieved more consecutive cycles <36 days, the number of ovulatory cycles increased (ROM‐1: 31% ovulatory vs. ROM‐2 and ROM‐3: 54% and 44% ovulatory, respectively) and the occurrence of relapse after recovery decreased (ROM‐1 relapse: 53% vs. ROM‐2 and ROM‐3 relapse: 15% and 22%, respectively).

**Conclusion:**

Multiple eumenorrheic cycles may be necessary to observe a significant increase in estrogen exposure, ovulation, and a decrease in relapse after recovery.

## INTRODUCTION

The prevalence of severe menstrual disturbances, including secondary functional hypothalamic amenorrhea (FHA) and oligomenorrhea, among female athletes has been reported to be as high as 60%.^1^ Secondary FHA is the absence of a menstrual period for at least 3 consecutive months (≥90 days) after experiencing menarche; oligomenorrhea, which has recently been termed “abnormal uterine bleeding (AUB‐infrequent)”,^2^ is characterized by long, irregular menstrual cycles of 36 to 90 days.^3^ In exercising females, menstrual disturbances are often due to energy deficiency, which occurs when energy intake is inadequate relative to energy expenditure. The primary treatment recommendation for menstrual disturbances resulting from energy deficiency is to increase energy intake over the course of at least 12 months, provided bone mineral density is not worsening or co‐occurring with an increased number of stress fractures.^3^, ^4^ FHA contributes to a sequelae of health consequences, including poor bone health,^5^, ^6^, ^7^ transient infertility,^3^ and physiological consequences associated with chronic hypoestrogenemia.^3^, ^8^, ^9^ Indeed, the Female Athlete Triad has been defined in the literature since 1992^10^ and updated since then^4^ to highlight the effects of energy deficiency on reproductive health and bone health. Indeed, the downstream effects of energy deficiency are well established to cause detrimental effects not only to bone density but also to bone quality.^6^, ^7^, ^11^ Further, energy deficiency and the associated ovarian suppression contribute to decrements in sport performance.^12^, ^13^ To be clear, Relative Energy Deficiency in Sport (REDs) is a commonly adopted model used to describe the effects of energy deficiency on a wide variety of health outcomes beyond the well‐established skeletal and reproductive effects, thereby building upon the Female Athlete Triad model; however, the use of this newer REDs model in sports medicine has grown faster than the scientific evidence.^14^

The recovery of menses (ROM) and the goal to improve the ovarian hormone environment is a top priority when treating severe menstrual disturbances in female athletes^4^; however, there is no standardized definition of ROM and the initial occurrence of menses following oligo‐/amenorrhea (Oligo/Amen) may not be commensurate with the return of ovulation and optimal ovarian function.^15^ Researchers have used varied definitions of ROM, including at least one menses,^15^, ^16^, ^17^ two consecutive menstrual cycles,^16^, ^18^, ^19^, ^20^, ^21^ three consecutive menstrual cycles or cycles of <36 days for at least 3 months,^22^, ^23^, ^24^ and three or more menses in 6 months.^25^, ^26^ Notably, a recent systematic review indicated that the majority of investigators that categorized ROM did not provide a definition of ROM (24 out of 34 studies).^27^ The inconsistency among ROM definitions in females with FHA makes it difficult for researchers to compare and accurately interpret physiological sequelae associated with ROM and poses challenges for clinicians who are monitoring menstrual recovery in their patients.

Clinically, serial hormone measurements when monitoring ROM in patients with Oligo/Amen are not feasible. As such, clinicians may rely on the occurrence of menses, or menstrual frequency, to determine whether recovery has occurred. Such a strategy is reasonable given the noninvasive and self‐report nature of the method; however, it is not well understood how increasing menstrual frequency translates to the reestablishment of adequate ovarian steroid (estrogen and progesterone) exposure and ovulation. Additionally, ROM may be short lived in some cases, with females experiencing repeated episodes of FHA or oligomenorrhea after initial menstrual cycle resumption (ie, relapse) given the episodic nature both of energetic status and menstrual cycles.^16^ Understanding the *quality* of menstrual recovery that is associated with increasing menstrual frequency, rather than simply focusing on the *occurrence* of recovery as has been previously reported, may allow researchers and clinicians to better identify meaningful ROM targets.

In the REFUEL randomized controlled trial (RCT), we explored the effectiveness of increasing energy intake (~350 kcal/day) on ROM over 12 months in exercising females with FHA and oligomenorrhea associated with energy deficiency. Daily urine samples were collected throughout the study to allow for detailed reporting of estrogen and progesterone exposure and ovulation that occurred in conjunction with menstrual recovery. Our intent‐to‐treat analyses indicated that although exercising females with severe menstrual disturbances randomized to increased energy intake were more likely to experience menses during the study,^15^ estrogen and progesterone did not significantly increase across the study to concentrations similar to that observed in an ovulatory reference group;^28^ this finding suggests that the simple occurrence of menses may not be associated with robust improvements in ovarian hormone secretion nor the occurrence of ovulation. Remaining to be explored in the REFUEL dataset is a detailed examination of the hormone exposure and ovulatory activity associated with ROM definitions of increasing rigor.

The overall purpose of this secondary analysis was to explore how varying degrees of menstrual recovery, defined by increasing the number of consecutive eumenorrheic cycles of <36 days, related to ovarian hormone concentrations and ovulation. Specifically, this paper aims to (1) describe ovarian hormone exposure and cycle quality (ovulatory vs. anovulatory) in relation to menstrual frequency, (2) compare ovarian hormone concentrations from baseline to the recovery cycle in females who achieved menstrual recovery based on varying definitions of ROM, and (3) describe the occurrence of relapse for varying definitions of ROM. We hypothesized that as the definitions of the ROM advance from one cycle of <36 days to two or three consecutive cycles of <36 days, indicating improvements in the consistency and regularity of menstrual cycles, the concentration of ovarian hormones and evidence of ovulation will increase, and the occurrence of relapse will decrease. The results of these analyses can be clinically translated to provide appropriate menstrual frequency targets for exercising females with menstrual disturbances to achieve ROM associated with ovarian steroid hormonal recovery.

## MATERIALS AND METHODS

### Study design

This study is a secondary analysis of data from the REFUEL RCT (clinicaltrials.gov #NCT00392873), which explored the influence of 12 months of increased energy intake on energetic status, reproductive function and menstrual regularity, and bone health in exercising females with Oligo/Amen. Specific methodological details of the RCT have been previously published,^29^ and the primary RCT outcomes have been reported.^15^, ^28^ Only participants who were randomized to the intervention group (Oligo/Amen+Cal), which increased energy intake for the duration of the 12‐month intervention, were used in these secondary analyses. The REFUEL study was conducted at two sites, the University of Toronto (2006–2008) and Pennsylvania State University (2008– 2014) with approval from the Research Ethics Board of the University of Toronto and the Institutional Review Board of Pennsylvania State University, respectively.

### Eligibility criteria

Eligibility criteria for the study were (1) age 18–35 years and female gender, (2) body mass index 16–25 kg/m^2^, (3) good health as determined by medical exam, (4) no chronic illness, (5) ≥3 hours/week of purposeful exercise (both recreational and competitive athletes were eligible), (6) nonsmoker, (7) not currently dieting, (8) no hormonal therapies for the past 6 months, (9) no current clinical diagnosis of eating or psychiatric disorder, (10) not pregnant or lactating or planning a pregnancy, (11) no medication use that would alter metabolic or reproductive hormone concentrations, and (12) no other contraindications to study participation. To rule out endocrine or metabolic diseases as a cause of the Oligo/Amen, a fasting blood sample was collected during the screening phase to assess concentrations of follicle‐stimulating hormone (FSH), luteinizing hormone (LH), estradiol, prolactin, thyroid‐stimulating hormone, thyroxine, total and free testosterone, and dehydroepiandrosterone sulfate.

### Nutritional intervention

The nutritional intervention consisted of an energy intake prescription that was 20%–40% above each participant's baseline energy requirements.^15^, ^29^ A clinical dietician counseled participants in the Oligo/Amen+Cal group to increase food intake via energy bars (220–300 calories) and premeasured servings of nuts that were supplied by the study team or via alterations to their own diet. We also asked participants to maintain their baseline habitual exercise throughout the study.^29^

### Determination of Baseline Menstrual Status

We determined menstrual status using information from medical history, endocrine measures (thyroid‐stimulating hormone, thyroxine, prolactin, estradiol, FSH, LH, LH/FSH ratio, total testosterone, sex hormone binding globulin, free androgen index, human chorionic gonadotropin), physical exam (which included an evaluation of hirsutism and acne), diet and exercise history and presence/absence of current eating disorder, and self‐reported menstrual status corroborated by daily urinary hormone profiles of estrone‐1‐glucuronide (E1G), pregnanediol glucuronide (PdG), and LH for a 28‐day monitoring period (amenorrheic) or a complete menstrual cycle (oligomenorrheic) at baseline.^30^ Criteria for assigning participants as amenorrheic were the self‐report of no menses for at least 3 months prior to the study and a suppressed ovarian hormonal profile with no evidence of menses during the baseline monitoring period. Criteria for assigning participants as oligomenorrheic were the self‐report of one or two menses in the past 3 months or less than seven menses in the past 12 months, or a menstrual cycle 36–89 days in length during the baseline period.^30^, ^31^ Additionally, a participant was also considered oligomenorrheic if her self‐reported menstrual history or baseline menstrual cycle indicated irregular menstrual cycles.^15^ We assessed the occurrence of menses throughout the study using self‐report corroborated by daily urinary reproductive hormone assessments and blinded determinations of menstrual function by two experts.^31^

### Definitions of menstrual recovery

We defined ROM in four ways to demonstrate advancing degrees of recovery. The four definitions are as follows:

Simple ROM: This definition was applied only to female athletes who were amenorrheic at baseline, and ROM occurred when at least one menses was experienced during the intervention.

The next three definitions applied to both amenorrheic and oligomenorrheic participants.

ROM‐1 (≥1 cycle): ROM occurred when exercising females had at least *one* menstrual cycle <36 days.

ROM‐2 (≥2 cycles): ROM occurred when exercising females had at least *two* consecutive menstrual cycles <36 days.

ROM‐3 (≥3 cycles): ROM occurred when exercising females had at least *three* consecutive menstrual cycles <36 days.

### Definition of relapse

We defined relapse as experiencing an episode of amenorrhea or oligomenorrhea during the intervention after satisfying any of the four menstrual recovery definitions.

### Classification of menstrual cycles

We classified menstrual cycles during the study according to ovulatory status, cycle length, and concentrations of the urinary metabolites (E1G, PdG, LH). The “recovery” cycle for each ROM definition was defined as the menstrual cycle that allowed the criteria to be met (eg, for ROM‐1, the first cycle <36 days; for ROM‐2, the second cycle <36 days; etc.).

We initially classified cycles into 10 different categories based on the hormonal profile of the cycle (optimal ovulatory, luteal phase defect with a short or inadequate luteal phase or both, anovulatory, oligomenorrheic with or without ovulation, amenorrheic, short ovulatory or anovulatory).^29^ An optimal ovulatory cycle was characterized by a cycle length ≤ 35 days and optimal reproductive hormone concentrations (E1G peak >35 ng/mL, PdG peak >5 μg/mL, LH >25 mIU/mL) to differentiate it from an ovulatory cycle with a subtle menstrual disturbance, that is, a luteal phase defect.^31^, ^32^, ^33^ Menstrual cycles with a luteal phase defect were ovulatory but demonstrated a short luteal phase (<10 days), an inadequate luteal phase (PdG: peak between 2.5 and 5 μg/mL and/or 3‐day sum (PdG peak±1d sum of <10 μg/mL), or both a short and inadequate luteal phase.^31^, ^32^, ^33^ We then collapsed classifications based on ovulatory status and cycle length to create four main classifications: (1) eumenorrheic ovulatory, (2) eumenorrheic anovulatory, (3) oligomenorrheic, and (4) amenorrheic (Table 1).

**TABLE 1:** Description of menstrual cycle classifications.

Menstrual cycle category	Criteria^a^	Cycle types
Ovulatory	E1G: > 35 ng/mL PdG: peak >2.5 μg/mL LH: >25 mIU/mL within 5 days after the E1G peak Cycle length ≤ 35 days	Optimal ovulatory Luteal phase defects; short and/or inadequate luteal phase Short ovulatory .
Anovulatory	E1G: suppressed (< 35 ng/mL) PdG: peak <2.5 μg/mL LH: no peak (<25 mIU/mL) Cycle length ≤ 35 days	Anovulatory Short anovulatory
Oligomenorrheic	Cycle length 36–89 days	Oligomenorrheic ovulatory Oligomenorrheic anovulatory
Amenorrheic	E1G: suppressed (< 35 ng/mL) PdG: suppressed (<2.5 μg/mL) LH: no peak (< 25 mIU/mL) Cycle length ≤ 35 days	Amenorrheic

Abbreviations: E1G, estrone‐1‐glucuronide; LH, luteinizing hormone; PdG, pregnanediol glucuronide.

^a^ PdG criteria applied to the luteal phase.

### Urinary reproductive hormone analysis

A detailed description of E1G, PdG, and LH measurements in urine samples has been previously published.^29^ To determine estrogen and progesterone exposure for a given menstrual cycle (if eumenorrheic or oligomenorrheic) or 28‐day monitoring period (if amenorrheic), we calculated area under the curve (AUC) for E1G and PdG using Kaleidagraph Software (Synergy Software, Reading, PA, USA). We also calculated mean E1G and PdG concentrations across the cycle.^29^ All urine samples were corrected for specific gravity using a hand refractometer (NSG Precision Cells, Farmingdale, NY) to account for hydration status,^34^ which has been reported to perform as well as creatinine correction for adjusting urinary hormone concentrations.^34^

We used microtiter plate competitive enzyme immunoassays to E1G and PdG, as previously published.^29^ The E1G (R522‐2) and PdG (R13904) assays use a polyclonal capture antibody supplied by Coralie Munro, University of California (Davis, CA). The interassay coefficients of variation for high and low internal controls for the E1G assay are 12.2% and 14.0% respectively. The PdG intra‐ and interassay variability was determined in house as 13.6% and 18.7%, respectively.^31^, ^35^

We measured urinary LH using a coat‐a‐count immunoradiometric assay (Siemens Healthcare Diagnostics, Deerfield, IL). The sensitivity of the LH assay is 0.15 mIU/mL. The intra‐ and interassay coefficients of variation were 1.6% and 7.1%, respectively.

### Missing urine samples

We applied the following rules when missing samples were encountered: (1) if ≤5 consecutive days were missing, the missing days were estimated using the data of the surrounding days; (2) if a cycle was missing >10 consecutive days anywhere in the cycle, the AUC and mean for E1G and PdG were not calculated or included in data analyses (occurred in one cycle); (3) if ≥5 days were missing between days 12–20 of the cycle or during the follicular phase, E1G AUC and mean were not calculated or included in data analyses (occurred in 2 cycles); (4) if ≥5 days were missing between days 18 to end of the cycle, PdG AUC and mean were not calculated or included in data analyses; and (5) if the missing days included the dates when the suspected E1G or PdG peak occurred, the cutoff for not reporting E1G and PdG AUC and mean was lowered to ≥4 missing days (occurred in 1 cycle). Consequently, either E1G or PdG data for 4 menstrual cycles (6% of the 63 total cycles analyzed for this paper) were not included in the analyses.

### Statistical analysis

We examined the proportion of exercising females who achieved ROM or experienced relapse after ROM, within each ROM definition and according to baseline menstrual status (amenorrhea or oligomenorrhea). We also assessed the proportion of recovery cycles that were ovulatory versus anovulatory based on baseline menstrual status.

After assessing data for normality using Shapiro–Wilk tests, we conducted paired *t*‐tests (normally distributed variables) or Wilcoxon signed rank tests (nonnormally distributed variables) to compare estrogen and progesterone exposure between a baseline menstrual cycle or monitoring period and the recovery menstrual cycle in participants who achieved ROM for ROM‐1, ROM‐2, and ROM‐3. Due to the variability in recovery cycle length, we were not able to compare ovarian hormone exposures from baseline to recovery for participants who achieved ROM‐simple. Continuous data were reported as mean ± SEM and the significance level was *α* < .05. We used IBM SPSS Statistics for Windows (Version 27.0, Armonk, NY, USA: IBM Corp.) for analyses.

## RESULTS

**FIGURE 1:**
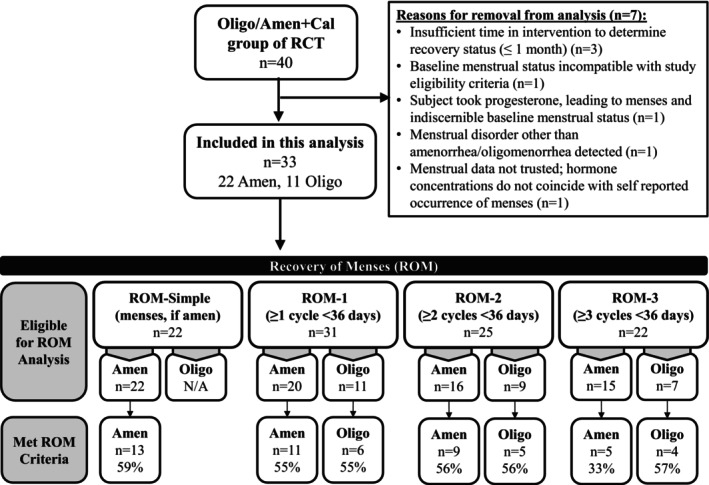
Flow chart of participants, which includes reasons for exclusion from analysis, recovery of menses definition eligibility, and number of participants achieving the ROM criteria for each definition. Percent indicates the percentage of the sample included in the analysis who met criteria for each ROM definition for ROM‐1, ROM‐2, and ROM‐3. Amen, Amenorrheic at baseline; Oligo, Oligomenorrheic at baseline; Oligo/Amen+Cal, Intervention group of females with oligo‐/amenorrhea at baseline who increased energy intake; RCT, randomized controlled trial; ROM: recovery of menses.

After the 4‐week baseline period of the RCT, we randomized 40 exercising females with menstrual cycle disturbances to the Oligo/Amen+Cal group. Seven of the 40 exercising females from the Oligo/Amen+Cal group were excluded from the current analyses due to the inability to discern menstrual recovery (specific reasons are provided in Figure 1), leaving 33 exercising females eligible for inclusion in these analyses. (A detailed figure of the entire RCT enrollment and dropout is provided in a previous publication that reported the primary menstrual recovery results of the study^15^). The duration of follow‐up in the intervention ranged from 1 to 12 months. Of the 33 exercising females included in the analyses, 27 females (82%) completed at least 3 months, 22 females (67%) completed at least 6 months, 21 females (64%) completed at least 9 months, and 16 females (48%) completed 12 months of the intervention. No single ROM definition included all 33 participants; for each definition, the exercising females that completed an adequate time in the study for a determination about ROM to be made for that specific definition were included in the pertinent analyses. Accordingly, 22 exercising females had the potential to meet ROM‐simple, 31 had the potential to meet ROM‐1 (≥1 cycle <36 days), 25 had the potential to meet ROM‐2 (≥2 cycles <36 days), and 22 had the potential to meet ROM‐3 (≥3 cycles <36 days) (Figure 1). Nineteen exercising females (19/33, 58%) recovered according to at least one of the ROM definitions. Table 2 provides the baseline characteristics for the females included in these analyses for any ROM definition.

**TABLE 2:** Baseline and intervention* characteristics of females included in the analyses (Oligo/Amen+Cal, *n* = 33) for any ROM definition.

	Baseline	Intervention
	Mean ± SEM	Mean ± SEM
Age (y)	21.5 ± 0.6	‐
Body weight (kg)	55.2 ± 1.1	56.9 ± 1.0
Height (cm)	164.4 ± 1.1	‐
Body mass index (kg/m^2^)	20.4 ± 0.4	21.0 ± 0.3
Age of menarche (y)	13.4 ± 0.2	‐
Duration of amenorrhea (days)	325 ± 76^a^	‐
Percentage of body fat (%)	23.6 ± 0.7	25.1 ± 0.7^c^
Fat mass (kg)	13.1 ± 0.5	14.3 ± 0.5^c^
Lean mass (kg)	40.0 ± 0.8	40.6 ± 0.9^c^
Energy intake (kcal/day)	1958 ± 99	2272 ± 142^d^
Exercise energy expenditure (kcal/day)	321 ± 48^b^	334 ± 52^e^
Menstrual cycle history	Median [interquartile range]	
Cycles in past 6 mo (#)	1 [2.0]	‐
Cycles in the past 12 mo (#)	4 [4.25]^b^	‐
Sport type	% (n)	
Endurance sports	82% (27)	‐
Aesthetic sports	6% (2)	‐
Weight class sports	6% (2)	‐
Ball sports	6% (2)	‐
Race	% (n)	
White	82% (27)	‐
Asian	15% (5)	‐

*Note*: Data are mean ± SEM for continuous variables, median [interquartile range] for ordinal variables, and % (*n*) for categorical variables.

Abbreviations: Oligo/Amen+Cal: intervention group of females with oligo‐/amenorrhea at baseline who increased energy intake; ROM: recovery of menses.

* Intervention refers to the last collected data point for each participant during/after the intervention.

^a^ *n* = 26;

^b^ *n* = 30;

^c^ *n* = 29;

^d^ *n* = 32;

^e^ *n* = 27.

### ROM‐simple recovery characteristics

For ROM‐simple, among the 22 exercising females with amenorrhea at baseline, 59% (*n* = 13/22) had at least one menses during the intervention (Figure 1). Sixty‐nine percent (*n* = 9/13) experienced a recovery cycle of normal length (<36 days, eumenorrheic); only two of these cycles were ovulatory. Three participants experienced an oligomenorrheic recovery cycle (36 to 89 days), two of which were ovulatory. One participant relapsed back to amenorrhea immediately following the initial menses. An additional participant experienced a relapse to amenorrhea later in the intervention; thus, of the 13 exercising females with amenorrhea at baseline who had at least one menses during the intervention, two (15%) relapsed to amenorrhea. Experiencing long cycles during the intervention was more common; approximately half (*n* = 7/13) of these females who recovered had at least one oligomenorrheic cycle during the intervention. Recovery characteristics for this ROM definition are described in Table 3.

**TABLE 3:** ROM‐simple (females with amenorrhea only, *n* = 13) recovery cycle characteristics.

Recovery cycle	% (*n*)	Cycle length (mean ± SEM)
Eumen Ov	15% (2)	32.5 ± 1.5
Eumen Anov	54% (7)	30.0 ± 1.8
Oligo	23% (3)	42.3 ± 3.2
Amen^a^	8% (1)	28.0^b^

*Note*: For the Oligo recovery cycles, 2 were ovulatory and 1 was anovulatory.

Abbreviations: Amen, amenorrheic; Eumen Anov, eumenorrheic anovulatory; Eumen Ov, eumenorrheic ovulatory; Oligo, oligomenorrheic; ROM, recovery of menses.

^a^ A recovery cycle was amenorrheic if a female had one menses followed by an episode of amenorrhea (no menses for >90 days).

^b^ Length of amenorrheic monitoring period.

### Menstrual recovery according to ROM‐1, ROM‐2, and ROM‐3

Fifty‐five percent of amenorrheic (*n* = 11/20) and oligomenorrheic (*n* = 6/11) exercising females met criteria for ROM‐1, 56% of amenorrheic (*n* = 9/16) and oligomenorrheic (*n* = 5/9) exercising females met criteria for ROM‐2, and 33% of amenorrheic (n = 5/15) and 57% of oligomenorrheic (*n* = 4/7) exercising females met criteria for ROM‐3 (Figure 1).

### Recovery cycle classifications for ROM‐1, ROM‐2, and ROM‐3

**FIGURE 2:**
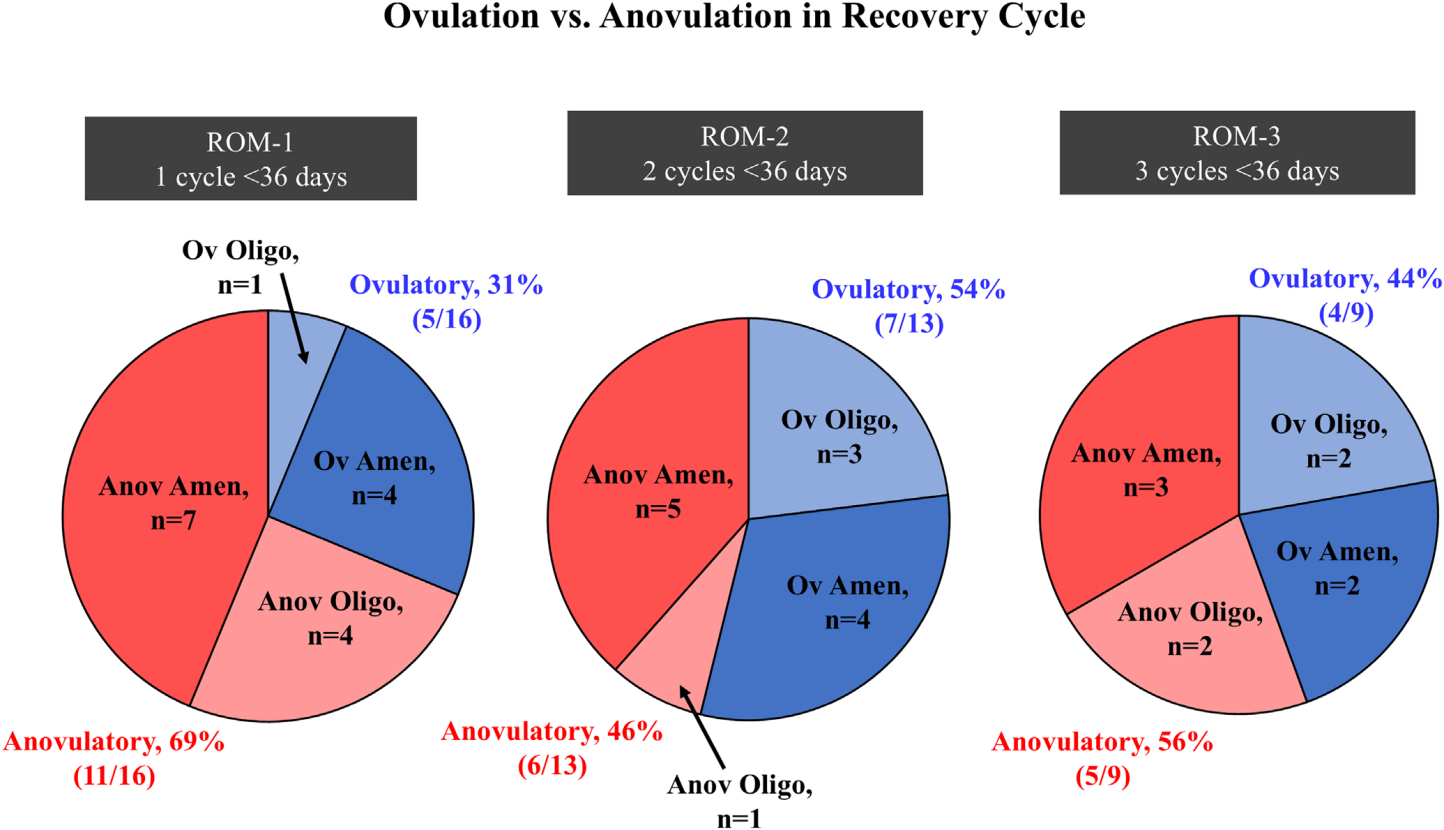
In blue, proportion of exercising females who recovered who had an ovulatory recovery cycle, as defined by E1G concentration > 35 ng/mL, a LH surge >25 mIU/mL within 5 days after the E1G peak, and a PdG peak in the luteal phase >2.5 μg/mL. In red, proportion of exercising females who recovered that had an anovulatory recovery cycle, as defined by estrone‐1‐glucuronide concentration < 35 ng/mL, a LH surge <25 mIU/mL, and a PdG peak in the luteal phase <2.5 μg/mL. Darker blue and red sections represent females with amenorrhea and lighter blue and red sections represent females with oligomenorrhea. For one female with oligomenorrhea, the recovery menstrual cycle for ROM‐1 and ROM‐2 could not be classified due to missing urine samples. Amen, amenorrhea; Anov, anovulatory; E1G, estrone‐1‐glucuronide; LH, luteinizing hormone; Oligo, oligomenorrhea; Ov, ovulatory; PdG, pregnanediol glucuronide; ROM, recovery of menses.

The percentage of ovulatory recovery cycles increased from 31% for ROM‐1 to 54% for ROM‐2 and 44% for ROM‐3 (Figure 2). Regardless of the ROM definition used (ROM‐1, ROM‐2, or ROM‐3), a minority of recovery cycles in exercising females who were amenorrheic at baseline were ovulatory (36 to 44%). In athletes with oligomenorrhea at baseline, only 20% of recovery cycles for ROM‐1 were ovulatory, but the proportion of ovulatory cycles increased to 75% of recovery cycles for ROM‐2 and 50% for ROM‐3. During the entire intervention, 54% (*n* = 7/13) of exercising females with amenorrhea at baseline and 50% (*n* = 3/6) of females with oligomenorrhea at baseline experienced at least one ovulatory cycle; of these ovulatory cycles, 84% (*n* = 26/31) had a luteal phase defect.

### Estrogen and progesterone exposure from baseline to recovery cycle for ROM‐1, ROM‐2, and ROM‐3

Because measurements of E1G/PdG AUC and mean are indexed to menstrual cycle length, baseline E1G/PdG exposure in amenorrheic exercising females represent a 28‐day monitoring period; whereas, in females with oligomenorrhea, baseline E1G/PdG exposure was determined based on a complete menstrual cycle prior to the intervention. For the females with oligomenorrhea included in the analysis of ROM‐1 and ROM‐2, average baseline cycle length was 33 ± 5 days. For the females with oligomenorrhea included in the analysis of ROM‐3, average baseline cycle length was 35 ± 7 days. Recovery cycle E1G/PdG AUC and mean are representative of a complete menstrual cycle, as described in the methods.

**FIGURE 3:**
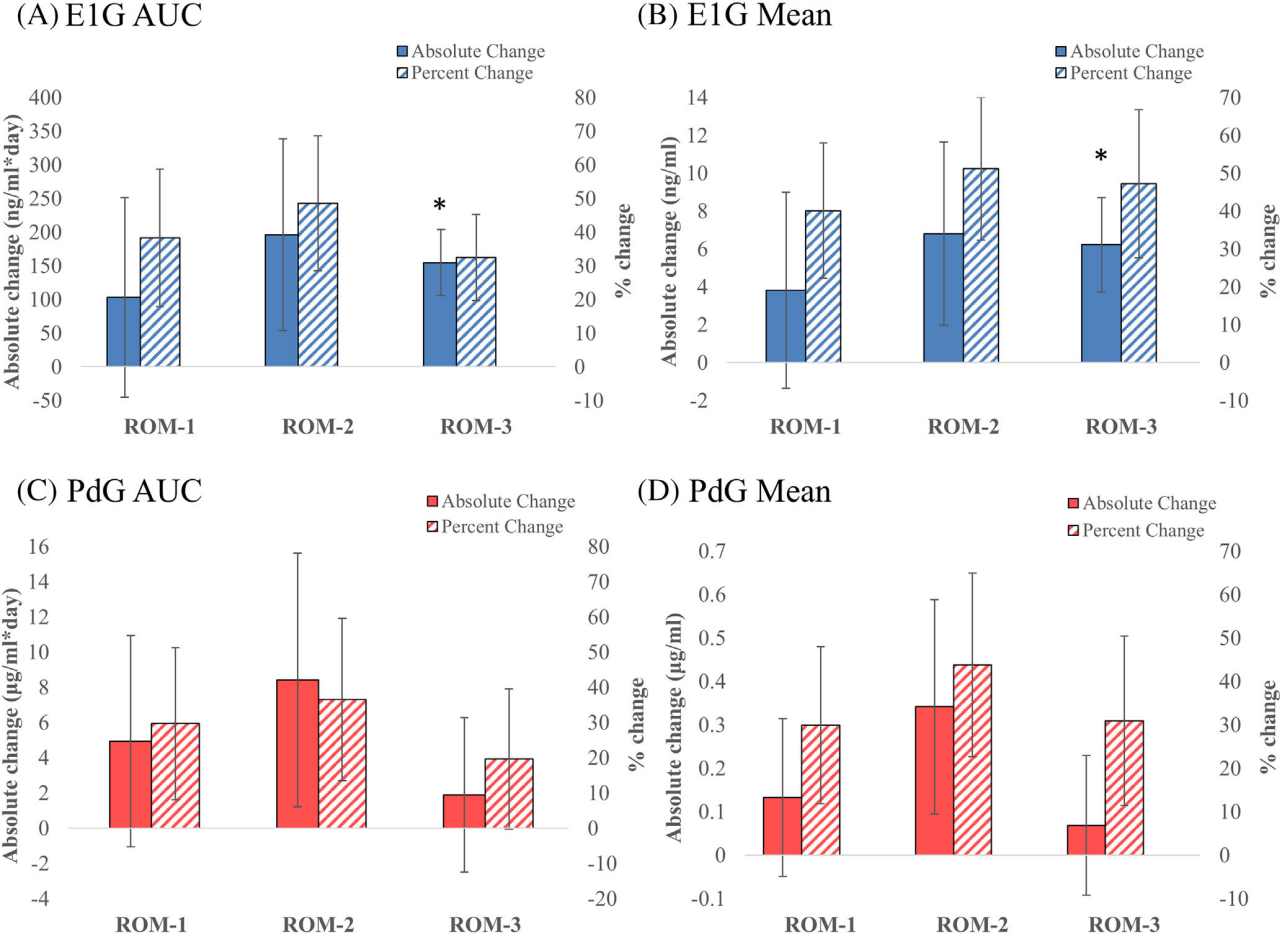
Absolute and percent change in E1G and PdG exposure from the baseline menstrual cycle/monitoring period to the menstrual cycle that allowed the recovery definition to be met. **A** depicts the results for E1G AUC, and **B** depicts the results for E1G mean. **C** depicts the results for PdG AUC, and **D** depicts the results for PdG mean. Solid bars depict absolute change, and striped bars depict percent change. Paired *t*‐test results *p < .05 absolute difference from baseline. ROM‐1 (≥1 cycle <36 days, *n* = 16); ROM‐2 (≥2 cycles <36 days, *n* = 13); ROM‐3 (≥3 cycles <36 days, *n* = 9). For one athlete with oligomenorrhea, the recovery menstrual cycle for ROM‐1 and ROM‐2 could not be analyzed due to missing urine samples. AUC, area under the curve; E1G, estrone‐1‐glucuronide; PdG, pregnanediol glucuronide; ROM, recovery of menses.

Figure 3 depicts the absolute and percent change in E1G and PdG AUC and mean concentration from baseline to the recovery menstrual cycle. There were no differences in E1G and PdG AUC and mean concentration for the recovery menstrual cycle compared with the baseline menstrual cycle/monitoring period for females who achieved ROM‐1 (*p* ≥ .472, Cohen's *d* = −0.2) or ROM‐2 (*p* ≥ .151, Cohen's *d* = −0.3). However, in those who achieved ROM‐3, estrogen exposure significantly increased from baseline to the recovery cycle (E1G AUC *p* = .014, Cohen's d = −1.05; E1G mean *p* = .037, Cohen's *d* = −0.83); progesterone exposure did not change (*p* = .68). The increase in estrogen exposure was not likely due to an increase in cycle length, as the length of the ROM‐3 recovery cycle (28 ± 2 days) and baseline cycle/monitoring period (31 ± 3 days) were similar (*p* = .59).

Analyzing females with amenorrhea and oligomenorrhea separately revealed that E1G AUC was significantly greater in the recovery cycle for ROM‐3 compared with the baseline monitoring period in females with amenorrhea (*p* = .011, Cohen's *d* = −1.99) but not in females with oligomenorrhea (*p* = .355, Cohen's d = −.55); menstrual cycle length was similar between the baseline monitoring period (28 ± 0 days) in females with amenorrhea and their recovery menstrual cycle (30 ± 2 days) (*p* = .48). PdG mean was significantly greater in the recovery cycle for ROM‐1 compared with the baseline cycle in females with oligomenorrhea (*p* = .043, Cohen's d = −0.89), despite cycle length being significantly shorter in the recovery cycle (26 ± 3 days) compared with the baseline cycle (33 ± 5 days) (*p* = .042).

### Relapse from ROM‐1, ROM‐2, and ROM‐3

**FIGURE 4:**
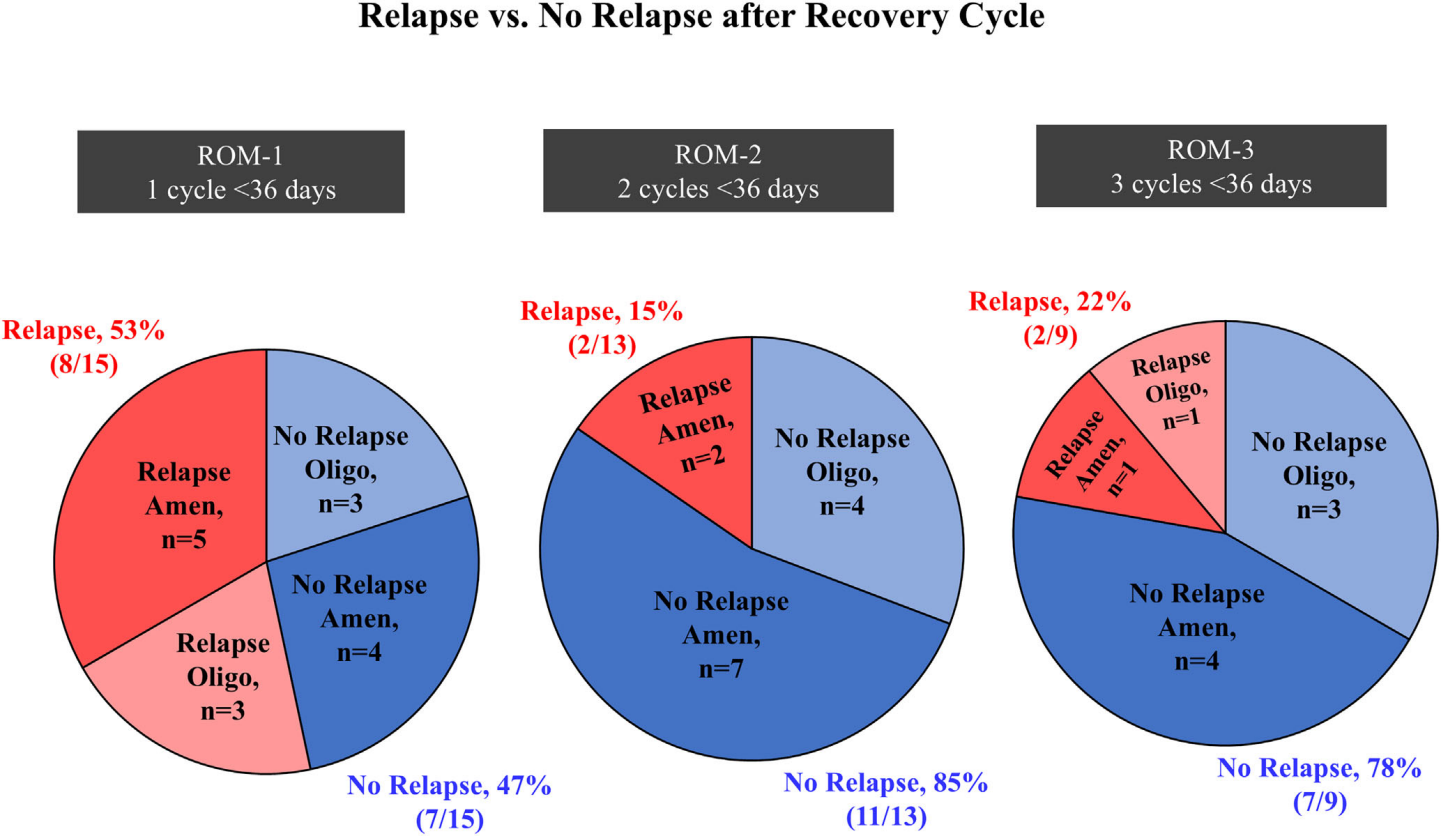
Proportion of females who experienced relapse to oligo‐/amenorrhea immediately after achieving recovery for that definition (in red) compared with proportion of females who experienced no relapse to oligo‐/amenorrhea immediately after achieving recovery for that definition (in blue) for each ROM definition. [Correction added on 18 May 2026 after first online publication: The previous statement has been revised.] Darker blue and red sections represent females with amenorrhea and lighter blue and red sections represent females with oligomenorrhea. Relapse data for three females could not be included (ROM‐1, *n* = 2; ROM‐2, n = 1) because the recovery cycle was the last cycle of study participation (ie, the participant was not observed beyond the recovery cycle to determine if relapse occurred or not.) Amen, amenorrhea; Oligo, oligomenorrhea; ROM, recovery of menses.

The proportion of exercising females who experienced a relapse episode of amenorrhea or oligomenorrhea after achieving ROM is provided in Figure 4. Among exercising females who met the ROM criteria for ROM‐1, 53% experienced an immediate relapse to amenorrhea or oligomenorrheic cycles. As the exercising females achieved more consecutive cycles (advancing from ROM‐1 to ROM‐2 and ROM‐3), the proportion of females experiencing immediate relapse decreased to 15% for ROM‐2 and 22% for ROM‐3. Among all females who experienced relapse, the majority (82%; 9/11) experienced a relapse to oligomenorrhea rather than amenorrhea. (Relapse data for 3 participants could not be included [ROM‐1, *n* = 2; ROM‐2, *n* = 1] because the recovery cycle was the last cycle of study participation, ie, the participant was not observed beyond the recovery cycle to determine if relapse occurred).

### Recovery characteristics based on time during intervention

Table 4 demonstrates the prevalence of menstrual recovery according to when it occurred during the study, that is, during the first half of the intervention (months 1 to 6) or the second half of the intervention (months 7 to 12). Regardless of the definition used, most exercising females who experienced menstrual recovery met the criteria within the first 6 months of the intervention. Table 4 also describes how many total ovulatory cycles occurred (among the 19 exercising females who recovered during the intervention) during the first half of the study versus the second half of the study. There were fewer eumenorrheic menstrual cycles during months 1–6 of the study (*n* = 36) compared to months 7–12 (*n* = 42). Regardless of the time during the study, the majority of eumenorrheic cycles were anovulatory. Of the ovulatory menstrual cycles, most of them had luteal phase defects (Table 4).

**TABLE 4:** Time course of recovery and occurrence of ovulatory menstrual cycles.

Recovery definition	Number of participants who recovered mo 1–6 % (*n*)	Number of participants who recovered mo 7–12 % (*n*)
ROM‐simple (menses in females with amenorrhea) (*n* = 13)	85% (11)	15% (2)
ROM‐1 (≥1 cycle <36 days) (*n* = 17)	100% (17)	0% (0)
ROM‐2 (≥2 cycles <36 days) (*n* = 14)	71% (10)	29% (4)
ROM‐3 (≥3 cycles <36 days) (*n* = 9)	56% (5)	44% (4)

All cycles < 36 days among athletes who recovered*	Number of cycles mo 1–6 (*n* = 36) % (*n*)	Number of cycles mo 7–12 (*n* = 42) % (*n*)
Ovulatory cycles	36% (13)	40% (17)
Optimal ovulatory cycles	6% (2)	7% (3)
Luteal phase defect cycles	31% (11)	33% (14)
Anovulatory cycles	64% (23)	60% (25)

Abbreviation: ROM, recovery of menses.

* Represents all cycles <36 days during the study for the 19 females who recovered according to any of the ROM definitions.

## DISCUSSION

The REFUEL study is the first RCT to examine the detailed effects of 12 months of increased energy intake on menstrual function in exercising females with amenorrhea or oligomenorrhea. To date, it is unknown how published ROM definitions that are based on menstrual frequency translate to ovulation or improvement in ovarian hormone secretion. This study provides valuable information of how menstrual frequency relates to ovulation and ovarian hormone exposure during nutritional efforts to reverse amenorrhea and oligomenorrhea.

The most important findings of this study include the following: (1) the majority of the cycles in the females who experienced a single onset of menses (ROM‐simple) during the intervention were anovulatory with poor and inadequate ovarian steroid exposure; (2) the findings were similar when menstrual recovery was categorized as a single cycle of <36 days (ROM‐1); (3) when menstrual recovery was categorized as at least 2 consecutive eumenorrheic cycles of <36 days (ROM‐2), a greater proportion of ovulatory menstrual cycles were observed but the cycles still failed to significantly improve estrogen exposure; and (4) estrogen exposure increased only among those females achieving the most advanced definition of menstrual recovery, that is at least three consecutive eumenorrheic cycles (ROM‐3) of <36 days. In other words, to observe a significant improvement in estrogen, the goal of menstrual recovery had to include at least three consecutive cycles of <36 days.

These findings provide important translational information for clinicians to provide to amen/oligomenorrheic exercising females attempting to recover menstrual health, that is, for resumption of menses to likely be associated with increased estrogen exposure, the goal must be to achieve at least three consecutive menstrual cycles of <36 days. Because our findings indicate that multiple menstrual cycles are likely necessary for improved estrogen exposure, the goal of multiple cycles is ideal as increased estrogen exposure should be the goal for a cycle to be considered healthy. Interestingly, however, progesterone exposure did not significantly increase even in the most advanced definition of ROM due to the relatively large proportion of anovulatory cycles that continued to occur. Indeed, although the proportion of ovulatory recovery cycles increased as the number of consecutive eumenorrheic cycles increased, anovulation persisted in a relatively large proportion of recovery cycles in this study. Even among exercising females who achieved three consecutive eumenorrheic cycles (ROM‐3), ovulation occurred in only about half (45%) of these recovery cycles.

We additionally found that as more consecutive menstrual cycles were achieved and recovery was more mature (advancing from ROM‐1 to ROM‐2 and ROM‐3), there were proportionally fewer females who experienced relapse. This finding underscores that simply experiencing a single menses event (ROM‐simple) or a single menstrual cycle of <36 days (ROM‐1) does not guarantee the continued recurrence of normal menstrual frequency, thus highlighting the importance of encouraging several consecutive menstrual cycles with ongoing long‐term monitoring of exercising females after the initial resumption of menses.

In the REFUEL study, “improvement” in menstrual function was demonstrated with a moderate increase in energy intake of approximately 350 kcal/day,^16^, ^36^, ^37^, ^38^ a volume of calories previously demonstrated to be associated with menstrual recovery in other studies.^36^, ^37^ We demonstrated that this volume of increase in energy intake per day was achievable, well received, not associated with detrimental psychological effects,^39^ and manageable for exercising females. Notably, however, few exercising females in the intervention achieved what we would consider to be “optimal” menstrual recovery, as defined by multiple consecutive ovulatory menstrual cycles during the 12‐month intervention period. Recovery menstrual cycles were commonly characterized by anovulation, although that percentage decreased as a female achieved more and more consecutive eumenorrheic cycles. Furthermore, when ovulation did occur, most of those cycles (84%) were characterized by a luteal phase defect, highlighting the persistent occurrence of subtle menstrual disturbances and suppressed ovarian hormone secretion among exercising females even when apparently regular menstrual cycles have resumed.

Few previous investigations of menstrual recovery have incorporated the sophistication of quantifying ovarian hormone exposure on a daily basis during the assessment period of recovery, in our case, 12 months. Indeed, a recent systematic review highlighted that menstrual recovery has not been consistently defined in the literature and, most often, ovarian hormone secretion has not been quantified during the recovery period.^27^ Of 34 published studies that evaluated the resumption of menses after Oligo/Amen in either female athletes or females with anorexia nervosa, only six studies (18%) evaluated the occurrence of ovulation during the recovery period.^27^ In agreement with our findings, multiple case studies have confirmed that dietary interventions successfully resulted in resumption of menses; however, ovulation was not observed.^16^, ^36^ On the other hand, a 6‐month intervention involving a 360 kcal/day dietary supplement in female athletes with Oligo/Amen, resulted in resumption of menses in all females (*n* = 8) and ovulation in all females but one.^37^ Notably, the sample sizes in these nutritional interventions were small, and to date, clear translatable targets for menstrual recovery among female athletes with Oligo/Amen are lacking, creating a challenge for clinicians.

As our findings and those of other investigators have demonstrated, the occurrence of a single menses or menstrual cycle as an indication of recovery does not indicate that ovulation or a significant improvement in ovarian hormone exposure has also occurred.^16^, ^36^, ^40^ Therefore, our findings provide translatable information for clinicians caring for exercising females with Oligo/Amen by demonstrating that the quality of menstrual recovery improves (ie, greater ovarian hormone exposure, greater likelihood of ovulation) as recovery progresses from one to two to three consecutive cycles of <36 days. Accordingly, exercising females should be monitored for several consecutive menstrual cycles when trying to achieve the onset of menses or more regular menstrual cycles after a period of amenorrhea or oligomenorrhea, respectively. Importantly, long‐term monitoring is necessary because several eumenorrheic menstrual cycles may be required to experience ovulation and decrease the risk of relapse.

A primary strength of this study is the thorough monitoring of menstrual function and detailed analysis of ovarian hormones for the duration of the intervention that permitted the careful characterization of menstrual cycles, ovarian hormone exposure, and the occurrence of relapse. We acknowledge that this study had a relatively high dropout rate, in part attributable to the high burden on participants of collecting daily urine samples for the study duration, frequent study visits, and other detailed requirements of the study, notwithstanding the requirement to increase energy intake. As such, the sample sizes for the most advanced definitions were small. The dropout rate was, however, comparable to other similar investigations; we experienced a 40% dropout by month 6 of our intervention compared with 30% dropout in an uncontrolled 6‐month nutritional intervention in exercising females.^41^ Another limitation of the study is the lack of ethnic diversity among the study participants, thereby limiting the generalizability of the results to females of various ethnic backgrounds. Finally, we acknowledge that psychosocial stress may contribute to menstrual cycle disturbances synergistically with energy deficiency,^42^ although perceived stress in this sample was similar to that observed in ovulatory counterparts.^39^

## CONCLUSION

The ongoing occurrence of ovulation and the associated “healthy” concentration of reproductive hormones are essential not only for reproductive health, including fertility, but also for many aspects of general health, including the maintenance of healthy bones, the maintenance of gluco‐metabolic health, cardiovascular and immune health and overall well‐being.^43^ As such, the attainment of regular menstrual function and ovulation should be viewed as a key aspect for overall health of females. This secondary analysis of the REFUEL RCT aimed to determine if the improvement of recovery of menses based on increasing the number of consecutive eumenorrheic menstrual cycles was associated with improvements in the ovarian hormone environment and quality of the menstrual cycle during recovery. In summary, multiple consecutive eumenorrheic cycles may be necessary to observe (1) a significant increase in estrogen exposure, (2) ovulation, and (3) a decrease in the occurrence of relapse after recovery. The occurrence of a single menses or eumenorrheic menstrual cycle does not indicate full menstrual recovery; although this is an important first step, continual monitoring of exercising females for more consecutive cycles is essential. Our findings also indicate that subtle menstrual disturbances, such as anovulation and luteal phase defects, persist during menstrual recovery, further highlighting the importance of continued follow‐up and intervention, as necessary, among female athletes. The time course and stimulus necessary for full menstrual recovery, as defined by ovulatory cycles without evidence of luteal phase defects, is currently unknown.

## AUTHOR CONTRIBUTIONS

Mary Jane De Souza and Nancy I. Williams conceptualized and designed the overall randomized controlled trial; acquired the funding, supervised and executed the trial, assisted with the design of this specific research question, and contributed to the writing and editing of the manuscript. Rebecca J. Mallinson was involved in conceptualization of this research question, data collection, data analysis and interpretation, and writing and editing the manuscript. Emily A. Ricker and Heather C. M. Allaway helped with data analysis and editing the manuscript. All authors read and approved the final version.

## FUNDING INFORMATION

This work was supported by the United States Department of Defense Congressionally Directed Medical Research Programs (CDMRP) under Grant number PR05431 (to Mary Jane De Souza and Nancy Williams). Additional research assistance provided by the Penn State Clinical Research Center was supported by the National Center for Advancing Translation Sciences, National Institutes of Health, through Grant UL1 TR002014.

## ETHICS STATEMENT

The study was conducted according to the guidelines of the Declaration of Helsinki and approved by the Institutional Review Boards of the Pennsylvania State University (protocol code 40851 and 10/1/2012) and University of Toronto. Informed consent using an approved consent document was obtained from all participants involved in the study.

## DISCLOSURE

The opinions and assertions expressed herein are those of the author(s) and do not reflect the official policy or position of the Uniformed Services University or the Department of Defense. The contents of this publication are the sole responsibility of the author(s) and do not necessarily reflect the views, opinions or policies of The Henry M. Jackson Foundation for the Advancement of Military Medicine, Inc. Mention of trade names, commercial products, or organizations does not imply endorsement by the U.S. Government. Mary Jane De Souza reports consulting fees from California Prune Board, Regeneron Pharmaceutical, and Coca Cola Company.

## Data Availability

The data that support the findings of this study are available from the corresponding author, Mary Jane De Souza, upon reasonable request.
